# Paradata analyses to inform population-based survey capture of pregnancy outcomes: EN-INDEPTH study

**DOI:** 10.1186/s12963-020-00241-0

**Published:** 2021-02-08

**Authors:** Vladimir Sergeevich Gordeev, Joseph Akuze, Angela Baschieri, Sanne M. Thysen, Francis Dzabeng, M. Moinuddin Haider, Melanie Smuk, Michael Wild, Michael M. Lokshin, Temesgen Azemeraw Yitayew, Solomon Mokonnen Abebe, Davis Natukwatsa, Collins Gyezaho, Seeba Amenga-Etego, Joy E. Lawn, Hannah Blencowe, the Every Newborn-INDEPTH Study Collaborative Group, Peter Byass, Stephen M. Tollman, Hagos Godefay, Joy E. Lawn, Peter Waiswa, Hannah Blencowe, Judith Yargawa, Joseph Akuze, Ane B. Fisker, Justiniano S. D. Martins, Amabelia Rodrigues, Sanne M. Thysen, Gashaw Andargie Biks, Solomon Mokonnen Abebe, Tadesse Awoke Ayele, Telake Azale Bisetegn, Tadess Guadu Delele, Kassahun Alemu Gelaye, Bisrat Misganaw Geremew, Lemma Derseh Gezie, Tesfahun Melese, Mezgebu Yitayal Mengistu, Adane Kebede Tesega, Temesgen Azemeraw Yitayew, Simon Kasasa, Edward Galiwango, Collins Gyezaho, Judith Kaija, Dan Kajungu, Tryphena Nareeba, Davis Natukwatsa, Valerie Tusubira, Yeetey A. K. Enuameh, Kwaku P. Asante, Francis Dzabeng, Seeba Amenga Etego, Alexander A. Manu, Grace Manu, Obed Ernest Nettey, Sam K. Newton, Seth Owusu-Agyei, Charlotte Tawiah, Charles Zandoh, Nurul Alam, Nafisa Delwar, M. Moinuddin Haider, Md. Ali Imam, Kaiser Mahmud, Angela Baschieri, Simon Cousens, Vladimir S. Gordeev, Victoria Ponce Hardy, Doris Kwesiga, Kazuyo Machiyama

**Affiliations:** 1 Institute of Population Health Sciences, Queen Mary University of London, London, UK; 2 Maternal, Adolescent, Reproductive & Child Health (MARCH) Centre, London School of Hygiene & Tropical Medicine, London, UK; 3 Department of Health Policy, Planning and Management, Makerere University School of Public Health, Kampala, Uganda; 4 Centre of Excellence for Maternal Newborn and Child Health Research, Makerere University, Kampala, Uganda; 5 Bandim Health Project, Bissau, Guinea-Bissau; 6 Research Centre for Vitamins and Vaccines, Statens Serum Institut, Copenhagen, Denmark; 7 Department of Clinical Research Open Patient data Explorative Network (OPEN), University of Southern Denmark, Odense, Denmark; 8 Kintampo Health Research Centre, Kintampo, Ghana; 9 Health Systems and Population Studies Division, icddr,b, Dhaka, Bangladesh; 10 Department of Medical Statistics, London School of Hygiene & Tropical Medicine, London, UK; 11 The World Bank, Washington DC, USA; 12 Dabat Research Centre Health and Demographic Surveillance System, Dabat, Ethiopia; 13 IgangaMayuge Health and Demographic Surveillance System, Makerere University Centre for Health and Population Research, Makerere, Uganda

**Keywords:** Survey, Paradata, Neonatal, Newborn, Answer correction type, Survey design

## Abstract

**Background:**

Paradata are (timestamped) records tracking the process of (electronic) data collection. We analysed paradata from a large household survey of questions capturing pregnancy outcomes to assess performance (timing and correction processes). We examined how paradata can be used to inform and improve questionnaire design and survey implementation in nationally representative household surveys, the major source for maternal and newborn health data worldwide.

**Methods:**

The EN-INDEPTH cross-sectional population-based survey of women of reproductive age in five Health and Demographic Surveillance System sites (in Bangladesh, Guinea-Bissau, Ethiopia, Ghana, and Uganda) randomly compared two modules to capture pregnancy outcomes: full pregnancy history (FPH) and the standard DHS-7 full birth history (FBH+). We used paradata related to answers recorded on tablets using the Survey Solutions platform. We evaluated the difference in paradata entries between the two reproductive modules and assessed which question characteristics (type, nature, structure) affect answer correction rates, using regression analyses. We also proposed and tested a new classification of answer correction types.

**Results:**

We analysed 3.6 million timestamped entries from 65,768 interviews. 83.7% of all interviews had at least one corrected answer to a question. Of 3.3 million analysed questions, 7.5% had at least one correction. Among corrected questions, the median number of corrections was one, regardless of question characteristics. We classified answer corrections into eight types (no correction, impulsive, flat (simple), zigzag, flat zigzag, missing after correction, missing after flat (zigzag) correction, missing/incomplete). 84.6% of all corrections were judged not to be problematic with a flat (simple) mistake correction. Question characteristics were important predictors of probability to make answer corrections, even after adjusting for respondent’s characteristics and location, with interviewer clustering accounted as a fixed effect. Answer correction patterns and types were similar between FPH and FBH+, as well as the overall response duration. Avoiding corrections has the potential to reduce interview duration and reproductive module completion by 0.4 min.

**Conclusions:**

The use of questionnaire paradata has the potential to improve measurement and the resultant quality of electronic data. Identifying sections or specific questions with multiple corrections sheds light on typically hidden challenges in the survey’s content, process, and administration, allowing for earlier real-time intervention (e.g.,, questionnaire content revision or additional staff training). Given the size and complexity of paradata, additional time, data management, and programming skills are required to realise its potential.

## Key findings

**Table:** 

**What is new?**	
• **What was known already:** Paradata are widely used in the field of survey methodology in high-income countries to monitor on-going fieldwork progress and identify issues with specific questions but have been little-used to date in low- and middle-income countries and for maternal, newborn, and child health data collection or research. • **What was done:** We analysed paradata from the EN-INDEPTH survey administered to 65,768 women of reproductive age in five countries. We assessed which question characteristics used to capture pregnancy outcomes affected duration of section completion, data correction rates, or were associated with multiple corrections and whether these differed by two maternity history modules (full pregnancy history (FPH) and full birth history (FBH+)).	
**What was found?**	
• **Corrections to questions were common:** affecting 83.7% of interviews, with a median of two questions corrected per interview and one correction per question when corrected (maximum of 28 corrections). 7.5% of the 3.3 million questions analysed had at least one correction. • **Simple one-time corrections most common:** accounting for 84.6% of all corrections. • **In variation in corrections by maternity history module:** number and type of corrections were similar between FPH and FBH+. • **In variation in corrections by question characteristics:** number and type of corrections were affected by question characteristics. The proportion of corrected questions was 3.3% higher for questions with notifications (9.8%) than for questions without notifications (6.5%). • **Duration of question completion:** was not affected by question characteristics (type, content, structure) or history type. Avoiding corrections has the potential to reduce interview duration and reproductive module completion by 0.4 min.	
**What next in measurement and research?**	
• **Measurement improvement now:** Paradata can be used to identify questions with multiple corrections, informing question editing or targeted training during and after survey completion. Encoding ranges and instant error notifications in the reproductive modules could reduce data missingness and prompt for timely data correction. Paradata analyses are skill- and time-consuming, but, if automatised, can be used for real-time data collection monitoring and data quality control. • **Research needed:** Studies could examine interviewer productivity and possible fatigue related to the length of the interview, the number of corrections, and correction types. The real-time dashboard monitoring and reporting systems using paradata could be evaluated in terms of associations with data quality and usefulness for survey management. Qualitative interviews with both respondents and interviewers would help to identify and verify factors affecting correction frequency to inform better questionnaire design and training adjustment.	

## Background

High-quality routine health data on maternal, newborn, and child health (MNCH) can be used to monitor, identify gaps, and take action to improve quality of care, optimise health system performance, and enable informed decision-making. Routine health management information systems vary in their completeness and quality across low- and middle-income countries (LMICs), and in many cases, are not able to provide the high-quality coverage data required for assessing and guiding health programmes [1, 2]. Household surveys, notably Demographic and Health Surveys (DHS) and Multiple Indicator Cluster Surveys (MICS), remain the primary sources of data for the outcome and coverage indicators for children and women for most low- and middle-income countries. However, despite existing quality control mechanisms in the survey process, data quality, including missingness, age displacement, and heaping, remains a challenge [3]. Optimising survey data efficiency and quality requires more information regarding the survey process and performance [4].

**Fig. 1:**
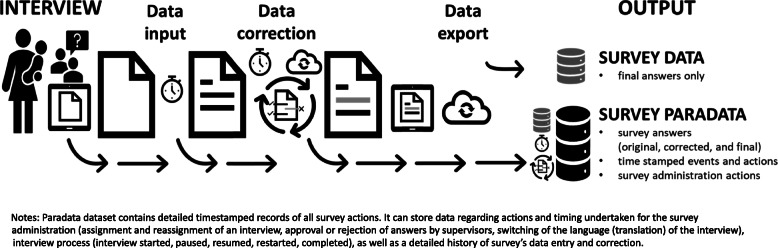
**Data collection cycle showing survey and paradata: EN-INDEPTH survey**

The shift from paper-based to computer-assisted personal interviewing (CAPI)-based data collection (e.g., using tablets and smartphones) has enabled inclusions of inbuilt validation and consistency checks, as well as a real-time review of collected data [5]. In addition to the main survey dataset (which contains only the final respondent’s answers), it is also possible to collect the survey’s paradata (Fig. 1). Paradata contain information on the process of how data for each observation in the main survey dataset was collected and include detailed timestamped records of all survey actions including survey administration, interview process, as well as a detailed history of all the survey’s data entry and correction [6, 7]. For example, paradata can show the order in which the questions were answered or corrected and reveal the content of deleted responses, which otherwise are not stored in the main survey dataset.

Even though paradata are widely used in the field of survey methodology in high-income countries [8], the use to date in LMICs and MNCH research has been limited. In household surveys, timestamped paradata can be used to monitor ongoing fieldwork progress and identify issues with specific questions or questionnaire sections based on the length of interviews or item response time [8]. Additional analyses can identify drivers behind item non-response and response time (e.g., survey locale; interviewer or respondent characteristics; survey’s content—questions’ type, nature, and structure) [9]. Paradata can also reveal determinants of data correction in relevant core survey questions during interviews as well as answer correction patterns (even though there is currently no agreed standardised terminology). Hence, paradata could lead to the overall improvements in data quality through targeted training [8] as well as improving questionnaire and survey design (structure and content) and survey implementation (process).

In this paper, we examine how paradata can be used to inform and improve questionnaire design and survey implementation in a large household survey collecting information on pregnancies and births using full pregnancy histories (FPH) and full birth histories with additional questions on pregnancy losses in the past 5 years (FBH+). This paper is one of a series of papers from the Every Newborn International Network for the Demographic Evaluation of Populations and Their Health (EN-INDEPTH) study in five Health and Demographic Surveillance System (HDSS) sites in sub-Saharan Africa and Asia.

This paper has three objectives:

1. To assess the differences in paradata timestamped entries between two reproductive modules (FPH and FBH+);
2. To determine whether question characteristics (type, nature, structure) affect the duration of section completion and answer correction rates;
3. To propose and test classification of answer correction types and determine whether they differ by two reproductive modules.

## Methods

### Overall EN-INDEPTH study design and data sources

**Fig. 2:**
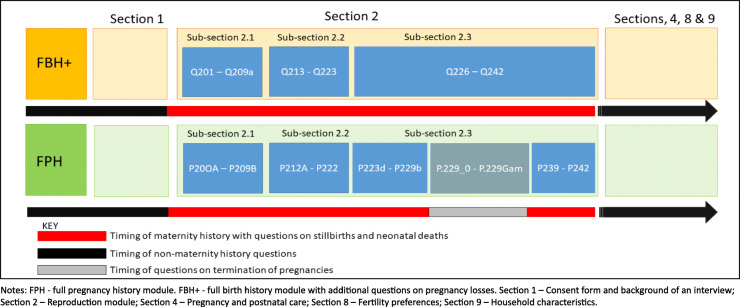
**Module structure for questions in the two arms of the EN-INDEPTH survey**

The EN-INDEPTH study aimed to compare two approaches of collecting maternity history (FPH and FBH+) to examine whether the two methods yield different estimates of stillbirth rates and neonatal mortality rates and to determine whether there is a difference in completion time for these two approaches. The study protocol and main findings can be found elsewhere [10, 11]. Briefly, the EN-INDEPTH survey reached 69,176 women of reproductive age in five HDSS sites (Bandim in Guinea-Bissau, Dabat in Ethiopia, IgangaMayuge in Uganda, Matlab in Bangladesh, and Kintampo in Ghana). Participants of the EN-INDEPTH study were randomly assigned (1:1) to be interviewed using a questionnaire containing either an FPH or an FBH+ module (section 2 in Fig. 2). The EN-INDEPTH study used the World Bank’s Survey Solutions CAPI/CAWI (computer-assisted web interviewing) data collection and management platform (hereafter Survey Solutions) [12] to collect face-to-face responses to the questionnaire (Additional file 1). The choice of the software and an overview of the data collection process and procedures are detailed elsewhere [13].

The analyses in this paper focus on the reproduction section (section 2) of the questionnaire only (Fig. 2). Section 2 contains three subsections. In subsection 2.1, women were asked to state their total lifetime number of liveborn children (FPH and FBH+) and a total number of pregnancy losses (FPH only). In subsection 2.2, women were asked for details about their lifetime pregnancies (FPH) or lifetime livebirths (FBH+) and answer a subset of questions for each instance. In subsection 2.3, women were asked in the FBH+ group about pregnancy losses in the past 5 years, while the FPH module contained an additional set of questions on termination of pregnancy (TOP), which we also included in the analyses (Additional file 1).

### Data processing

Paradata were exported in a tab-delimited format from the Survey Solutions platform [12], with each line corresponding to one recorded event (example in Additional file 2). Data from all sites were fully anonymised and required subsets of data extracted and merged using the R software [14]. We included only timestamped entries related to answers and corrections from section 2. We excluded entries that related to assigned but never conducted interviews and duplicate entries that resulted from updating questionnaire and software. All subsequent analyses were performed using Stata 16.1 [15].

### Methods by objective

#### Objective 1: To assess differences in paradata timestamped entries between two reproductive modules (FPH and FBH+)

Time taken to complete a question was estimated based on the difference between the timestamps of the previously answered question and the current one (based on answered questions order) and separate per observation (in case of parity > 1). For questions with corrections, the timestamp of the final answer was taken as the question’s timestamp. To exclude implausible values, but allow for multiple corrections during the module completion and/or switching between questionnaire sections during the interview [16], we restricted analyses of time taken to complete maternity history section of the survey to interviews lasting 0–180 min.

We categorised all questions by type, nature, and structure. The question types included single-select (e.g., ‘Was that baby a twin?’); multi-select (e.g., ‘Who assisted with the delivery of this baby?’); numerical computational (e.g., ‘How many children do you have?’); date-related (e.g., ‘What was the date of birth for this baby?’); and free-text input (e.g., ‘What is the name of your baby?’). There were three categories based on questions’ nature: two groups of potentially sensitive questions (death-related—relating to death and/or pregnancy loss, and TOP-related questions) and regular (non-sensitive) questions. Lastly, considering question structure, there were questions with built-in error notifications (e.g., displaying “value outside the range, please correct”), warnings appearing in capital red letters, or any other prompts for correction (e.g., when answers for age based on birth and age at last birthday did not match) and those without such notifications. Differences between FPH and FBH+ were evaluated using descriptive statistics and independent sample *t* test. Statistical significance level was defined at the 5% level.

#### Objective 2: To determine whether question characteristics affect the duration of section completion and answer correction rates

Differences in the duration of response time and proportion of corrections by question characteristics (type, nature, structure) were evaluated using descriptive statistics. A two-part model was used to analyse which question characteristics are associated with the likelihood of question correction (generalised linear model (GLM) with a binomial distribution and logit link) and the number of corrections (GLM with gamma distribution and log link function). Explanatory variables included question characteristics. Models were adjusted for respondent’s characteristics and location, with interviewer clustering accounted as a fixed effect. Statistical significance level was defined at the 5% level.

#### Objective 3: To propose and test classification of answer correction patterns and determine whether data correction patterns differ by two reproductive modules

In paradata, the process of data collection where all answers are entered and corrected is recorded as an ordered list of answers (sequence). To understand this process of data entry and correction better, we ordered all interviews based on the total number of questions asked during the interview and the number of answers (length of a sequence). We distinguished between original answers and corrections and visually inspected the resulting sequence index plot [17].

Whenever an answer to the same question has multiple corrections, these corrections can form a distinct pattern. For example, corrections can be single or multiple; the value of the original answer and the last correction may or may not match; correction entries may have identical or different values and may lead to missing data. As currently there is no classification of answer correction types, we developed and tested one using our survey data. We then used descriptive statistics to examine whether answer correction patterns vary by question characteristics and two reproductive modules.

Results are reported in accordance with STROBE Statement checklists for cross-sectional studies [18] (Additional file 3).

## Results

### Objective 1: To assess differences in paradata timestamped entries between two reproductive modules (FPH and FBH+)

#### Number of timestamped entries

We analysed 3.6 million timestamped entries corresponding to 3.3 million answered questions and their correction for 65,768 interviews, of which 52.1% related to FPH module (32,744 interviews), which by design contained more questions than the FBH+ reproductive module (33,024 interviews) (Table 1 and Additional file 4). Among all entries, 18.5% related to the pregnancy or birth history (sub-section 2.1), 66.2% to the roster (sub-section 2.2), and 15.3% to reproduction subsections of FPH and FBH+ reproductive modules (sub-section 2.3) (Fig. 2). The median number of timestamped answers per interview was 48 (52 and 45 for FPH and FBH+, respectively).

**Table 1:** Interview process details: number of timestamped entries, response time and corrections

Indicator	Overall			FPH			FBH+			***P*** value^**$**^
	Mean (SD)	Median	Range		Median	Range	Mean (SD)	Median	Range	
*N* timestamped entries per interview^a^	55.1 (33.4)	48	1–335	57.7 (34.9)	52	1–335	52.5 (31.5)	45	6–289	< 0.001
*N* questions answered per 1 interview	50.7 (29.6)	44	1–223	53.2 (31.1)	49	1–223	48.4 (27.8)	41	6–194	< 0.001
Response time per 1 interview^b^, min	10.8 (14.3)	7.3	0.06–179.9	11.4 (14.8)	7.8	0.06–179.9	10.3 (13.3)	6.9	0.23–179.8	< 0.001
Response time per 1 question^b^, min	0.4 (3.5)	0.08	0–179.9	0.4 (3.6)	0.08	0–179.9	0.4 (3.6)	0.07	0–179.9	
*N* corrected questions per 1 interview	3.8 (4.8)	2	0–112	3.9 (4.9)	3	0–112	3.6 (4.8)	2	0–110	< 0.001
*N* corrected question per 10 questions	0.8 (0.9)	0.6	0–29.5	0.8 (0.9)	0.6	0–29.5	0.8 (0.9)	0.62	0–20.4	< 0.01
*N* corrections per 1 interview	4.4 (6.2)	3	0–227	4.6 (6.3)	3	0–227	4.2 (6.1)	2	0–149	< 0.01
Time spent on correction per 1 interview, min	1.9 (10.2)	0.3	0–179.6	2.0 (10.2)	0.3	0–174.5	1.9 (10.2)	0.2	0–179.6	< 0.01
Time spent on correction per 1 question, min	0.6 (5.6)	0.08	0–179.9	0.6 (5.5)	0.08	0–179.8	0.6 (5.6)	0.08	0–179.9	
Response time per 1 interview, if all corrections avoided, min	9.4 (10.1)	6.9	0.06–179.5	9.9 (10.6)	7.4	0.06–179.5	8.8 (9.4)	6.5	0.2–179.4	< 0.001
Response time per 1 question, if corrections avoided, min	1.7 (4.5)	0.8	0.5–177.1	1.7 (4.5)	0.8	0.5–177.1	1.7 (4.5)	0.9	0.5–176.1	
*N* interviews, *n* (%)	65,768 (100.0)			32,744 (49.8)			33,024 (50.2)			
*N* interviews with at least 1 correction, n (%)	55,066/65,768 (83.7)			27,721/32,744 (84.6)			27,345/33,024 (82.8)			

Total percentages may not add up or exceed one hundred due to rounding up

*FPH* full pregnancy history module, *FBH+* full birth history module with additional questions on pregnancy losses

^$^*P* values for independent sample *t* test that compared means for two groups

^a^All timestamped entries, including answer corrections

^b^All answers, accounting for correction time

#### Type, nature, and structure of questions

FPH and FBH+ modules contain 98 and 66 possible uniquely formulated question/answer fields, respectively (Additional files 1 and 4). FPH reproductive module contains 52 single-select questions, 26 numerical computational, one date-related, and 17 free-text and two multi-select types of questions. FBH+ module contains 35 single-select questions, 28 numerical computational, one date-related, and two free-text and no multi-select types of questions. FPH has 18 questions related to death/pregnancy loss and 39 questions related to TOP (including country-specific questions). FBH has 27 questions related to pregnancy loss/death. The rest of the questions are regular (non-sensitive) by nature. A quarter of questions in FPH and about roughly a third in FBH+ have built-in error notifications.

Most of the timestamped entries related to single-select questions (66.6%), followed by numerical computational (32.1%), date-related (1.2%), and less than 0.03% being free-text and multi-select types of questions (Additional file 4). The proportion of timestamped entries per question type between modules was very similar. In terms of the questions’ nature, most of the timestamped entries were for regular questions (87.2%) and not related to the two groups of potentially sensitive questions (death and/or pregnancy loss, and TOP-related questions). In terms of structure, about a third of timestamped entries were for questions that had built-in error notifications, warnings, or other prompts for a correction.

#### The average duration of section and question completion

**Fig. 3:**
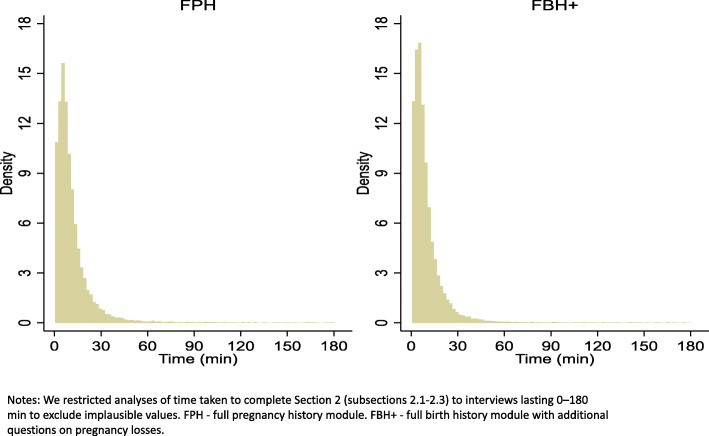
**Time to complete questions regarding maternity history (section 2) for the two survey modules (*N*** **= 60,871)**

The median number of questions answered per one interview was 44: 49 for FPH and 41 for FBH+, as FPH contained an additional set of TOP-related questions absent in FBH+. The median duration of section 2 completion was 7.3 min (Table 1, Fig. 3). The average time taken to complete the reproduction module was 1.1 min longer for the FPH (mean = 11.4 min) than the FBH+ (10.3 min). The median response time per question was around 0.1 min overall and for both modules.

#### Corrections per interview and questions

Overall, 83.7% of all interviews had at least one corrected answer to a question, slightly higher for FPH module than FBH+ (84.6% and 82.8%, respectively) (Table 1). The median number of corrected questions per interview was two, and a median number of corrections was three. The median time spent on corrections per one interview was 0.3 min (the mean time spent on correction was 0.1 min longer for FPH than FBH+), and the median time to correct one question was 0.08 min. Without corrections, the median response time per interview would be lower by 5.5% or 0.4 min.

### Objective 2: To determine whether question characteristics (type, nature, structure) affect the duration of section completion and answer correction rates

#### Duration of question completion

**Fig. 4:**
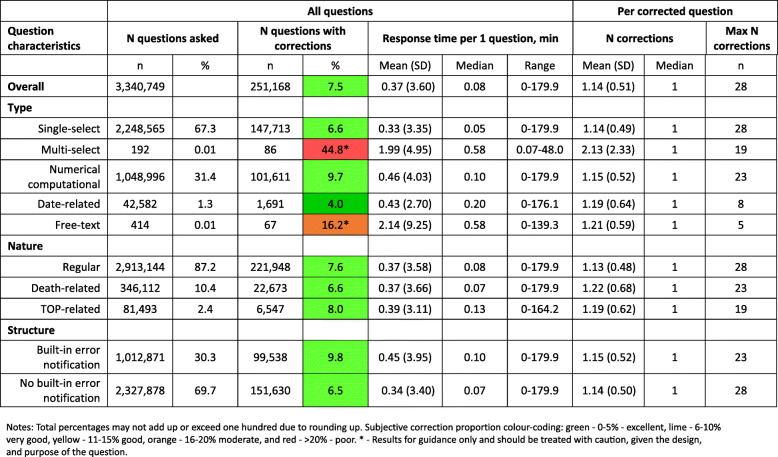
**Question corrections by question type, nature and structure**

The median response time per question type was longest for free-text and multi-select questions (0.6 min), followed by date-related (0.2 min), numerical computational (0.1 min), and single-select questions (0.05 min) (Fig. 4). The median response time only slightly varied per question’s nature, with the longest median response time for TOP-related questions (0.1 min). Questions with built-in error notifications had a median response time of 0.1 min compared with 0.07 min for questions with no built-in error notifications.

#### Proportion of corrections

Of all asked and answered questions, 7.5% had at least one or more corrections (Fig. 4, Additional file 5). Among all questions, the most frequently (by absolute number) corrected questions were single-select and numerical computational types of questions, regular questions, and questions without built-in notifications. However, the highest proportion of corrections within the question type group was multi-select questions (44.8%), followed by free-text (16.2%), numerical computational (9.7%), single-select (6.6%), and date-related (4%) questions. The proportion of corrections was similar based on the question’s nature (around 6–8%). The proportion of corrected questions was 3.3% higher for questions with notifications (9.8%) than for questions without notifications (6.5%).

Among corrected questions, the median number of corrections was one, regardless of question characteristics. The maximum number of corrections was the highest for single-select (*n* = 28) and numerical computational (*n* = 23) types of questions. In terms of questions’ nature, the maximum number of corrections was among regular questions (*n* = 28), followed by death-related questions (*n* = 23) and TOP-related questions (*n* = 19). Regarding the question structure, the maximum number of corrections was highest for questions with no built-in notifications (*n* = 28).

Based on the results of the regression analyses (Table 2, model 1), date question type (reference—single) and death-related questions (reference—regular) decreased the probability of making corrections. All other question characteristics increased the probability of making answer corrections when compared to their reference groups. Question characteristics (numeric, date, multi-select, death- and TOP-related) were positively associated with the number of corrections. Questions with notifications were negatively associated with the number of corrections. There was not enough evidence of an association between belonging to either of the two reproductive modules with either the probability of making a correction or the number of corrections.

**Table 2:** Question characteristics associated with answer correction probabilities and frequencies

Characteristics	Model (1)						Model (2)					
	Part 1 correction (yes/no)			Part 2 number of corrections			Part 1 correction (yes/no)			Part 2 number of corrections		
Variables	Coef	Robust Std. Err.	*p* value	Coef	Robust Std. Err.	*p* value	Coef	Robust Std. Err.	*p* value	Coef	Robust Std. Err.	*p* value
**Type, Ref (single)**												
Numerical computational	0.056	0.012	< 0.001	0.050	0.007	< 0.001	-0.138	0.014	< 0.001	0.026	0.008	0.001
Date-related	− 0.531	0.025	< 0.001	0.055	0.013	< 0.001	-0.411	0.028	< 0.001	0.072	0.015	< 0.001
Free-text	0.850	0.146	< 0.001	0.031	0.061	0.611	0.864	0.164	< 0.001	0.041	0.075	0.581
Multi-select	2.248	0.145	< 0.001	0.590	0.118	< 0.001	2.337	0.158	< 0.001	0.598	0.123	< 0.001
**Nature, Ref (regular)**												
Death-related	− 0.166	0.009	< 0.001	0.079	0.004	< 0.001	-0.187	0.011	< 0.001	0.078	0.005	< 0.001
TOP-related	0.205	0.015	< 0.001	0.040	0.007	< 0.001	0.316	0.017	< 0.001	0.055	0.008	< 0.001
**Structure, Ref (no notification)**												
Yes, with notification	0.400	0.012	< 0.001	− 0.041	0.007	< 0.001	0.486	0.014	< 0.001	− 0.026	0.008	0.001
**Module, Ref (FPH)**												
FBH+	0.015	0.009	0.101	− 0.003	0.003	0.248	0.012	0.009	0.169	− 0.003	0.003	0.333
**Constant**	− 2.661	0.007	< 0.001	0.125	0.002	< 0.001	-2.818	0.056	< 0.001	0.1465	0.016	< 0.001
Observations	3,340,189			250,608			2,247,142			152582		
R-squared	0.004			0.004			0.014			0.014		
Root MSE	0.263			0.511			0.250			0.494		

Model (1) unadjusted, model (2) adjusted for respondent’s characteristics (age, education, parity, wealth quintile) and location, with interviewer clustering accounted as a fixed effect. Both models accounted for clustering of individual responses within individual women (interview)

*FPH* full pregnancy history module, *FBH+* full birth history module with additional questions on pregnancy losses

After adjusting for respondent’s characteristics and location, with interviewer clustering accounted as a fixed effect (Table 2, model 2), all question characteristics remained significantly associated with the probability of making answer corrections when compared to their reference groups; however, the numeric type changed the direction of the association. Numeric, date, multi-select, and death- and TOP-related questions continued to be positively associated with the number of corrections, while the questions with notifications remained negatively associated with the number of corrections.

### Objective 3: To propose and test classification of answer correction types and determine whether they differ by two reproductive modules

#### Correction patterns

**Fig. 5:**
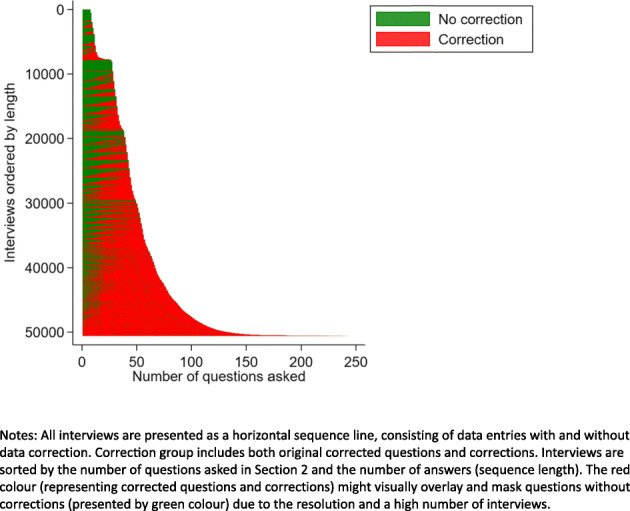
**Data collection and correction as a sequence index plot by the length of interview**

In line with our findings for objectives 1 and 2, the visual inspection of the sequence index plot (Fig. 5) showed that most of the interviews had corrections to answers. Only a smaller number of shorter interviews seemed to have no or a limited number of corrections. As the number of asked questions during the interview increased, so did the number of answer corrections. Based on the number of corrections per question, we identified 23 correction patterns, ranging from one to 28 corrections (Additional file 6). Most questions had single correction (89.0%), followed by multiple corrections (two and three corrections, 8.8% and 1.6%, respectively). The remaining 0.6% of questions had four and more corrections per question. We also observed that among these correction patterns, the original answer (first entry) sometimes matched the final answer correction (last entry), while for others, it did not. Some patterns consisted of either repetitive sequences of identical entries or a combination of different entries.

#### Correction types

By combining characteristics of answer correction process (single vs multiple corrections; first and last answer match vs do not match) and correction pattern (different vs identical entries), we developed a classification of answer correction types. We distinguished between eight possible answer correction types after accounting for possibly missing answers after corrections (Table 3). Building on the original terminology used by Elliot (1934) for cycles and pattern market behaviours and using elements of the original terminology for corrective waves (i.e., flat, zigzag) [19], we named correction types as no correction, impulsive, flat (simple), zigzag, flat zigzag correction, missing after correction, missing after a flat (or zigzag) correction, missing, or incomplete. Additionally, we distinguished between non-problematic (no correction, repetitive entry of identical values, or accidental mistake) and problematic (multiple corrections, first and last entry do not match, or missing after correction) correction types.

**Table 3:** Classification of answer correction type and possible explanations

Answer correction pattern (from first to final answer entry)	Multiple (> 1) corrections	First and last entry match	Correction type	Problematic	Possible explanation
A^a^	NO	YES	No correction	NO	n/a
A > A > A	YES	YES	Impulsive	NO	Multiple tapping (e.g., due to tablet’s lag response)
A > B^b^	NO	NO	Flat (simple) correction	NO	Accidental mistake, correction, following clarification or mistake
A > n^c^ > A	YES	YES	Zigzag correction	YES	Corrections after multiple additional clarifications and confirmation (e.g., change in responses) and changed back to the original answer
A > n > B	YES	NO	Flat zigzag correction	YES	Correction, following clarifications
A > …	NO	NO	Missing after correction	YES	Accidental mistake corrected after clarification, request to skip or not to record the answer
A > B > … or A > n > B > … or A > n > A > …	YES	NO	Missing after flat (zigzag) correction	YES	Clarification and consequent refusal to answer or request to skip or not to record the answer
…	NO	YES	Missing/incomplete	YES	Unknown

^a^stands for the original (first entered) answer

^b^represents a different answer in content than the original answer A

^c^represents a number of answer corrections between first and last answer in sequence

We tested our proposed classification using our survey data. Out of eight proposed answer correction types (Table 3), we observed only five, including no correction type (Table 4). Among answers with corrections, a flat (or simple) correction was the most frequent answer correction type (84.6%), followed by zigzag and flat zigzag corrections (5.3–5.5%), and the impulsive correction type (4.6%). As we did not treat the ‘Do not know’ as a missing answer and had no observations with truly missing answers, we did not observe the other four proposed answer correction types.

**Table 4:** Answer correction types per question type, nature, and structure

Question characteristics	*n*	Flat (simple)		Impulsive		Zigzag correction		Flat zigzag correction	
		*n*	%	*n*	%	*n*	%	*n*	%
**Overall**	250,608	212,057	84.6	11,438	4.6	13,288	5.3	13,825	5.5
**Type**									
Single-select	147,157	123,818	84.1	7613	5.2	10,124	6.9	5602	3.8
Multi-select	86	47	54.7	4	4.7	6	7.0	29	33.7
Numerical computational	101,607	86,850	85.5	3604	3.5	3080	3.0	8073	7.9
Date-related	1691	1290	76.3	213	12.6	76	4.5	112	6.6
Free-text	67	52	77.6	4	6.0	2	3.0	9	13.4
**Nature**									
Regular	221,447	188,670	85.2	9917	4.5	10,774	4.9	12,086	5.5
Death-related	22,628	17,871	78.9	1390	6.1	1905	8.4	1462	6.5
TOP-related	6533	5516	84.4	131	2.0	609	9.3	277	4.2
**Structure**									
Built-in error notification	99,532	85,244	85.6	3492	3.5	2979	3.0	7817	7.9
No built-in error notification	151,076	126,813	83.9	7946	5.3	10,309	6.8	6008	4.0

The flat correction type was the most frequently observed correction type (84.1%), irrespective of question type, nature, or structure (Table 4). A zigzag correction was the second most frequent type of correction for single-select questions (6.9%), while impulsive and flat zigzag correction types were the second most frequently observed correction types for date-related (12.6%) and numeric computational (33.7%) question types, respectively. Accounting for the question’s nature, a zigzag correction was the second most frequently observed correction type for death-related questions and TOP-related questions. For regular questions, the observed proportions were similar. Among questions with built-in notifications, the flat zigzag correction was the second most frequent type, while for questions with no built-in notifications, it was the zigzag correction.

## Discussion

This is the first study to examine the feasibility and usefulness of paradata to enhance household survey capture of pregnancy outcomes to inform the content, timing, process, and administration of questions. We delve further into our earlier findings on the lack of statistically significant differences in response times for FPH or FBH+ modules’ completion [11]. Around 84% of interviews had at least one correction to questions; however, most of them were simple one-time corrections. We identified four out of eight proposed answer correction types (impulsive, flat (simple) correction, zigzag correction, and flat zigzag correction) and found that question characteristics (type, content, structure) could affect the probability of making answer correction, be associated with the number of corrections, and vary in answer correction types. They remained to be significant predictors even after adjusting for respondent’s characteristics and location, with interviewer clustering accounted as a fixed effect. At the same time, the correction patterns based on the number of corrections per question and answer correction types were very similar between the two reproductive modules. The latter two were also not found to be significantly associated with the probability of making answer corrections and the number of answer corrections.

Even though the median number of corrected questions per interview and corrections per one question was relatively low (2 and 1), the maximum numbers of corrected questions and corrections per question were high (110 and 28, respectively). Not only does this add to the duration of section completion (as shown in our results), and ultimately to interview duration, it could also potentially shed light on questions that are poorly understood or misunderstood by either respondent or interviewer. For example, in our survey, the highest proportion of corrections was among the numerical computational (9.7%) and TOP-related (8.0%) questions. At the same time, even higher proportions of corrections were observed for multi-select questions (44.8%) and free-text (16.2%) questions. They also had the strongest association with the probability of making answer corrections. However, these results should be treated with caution as these questions constitute less than 0.1% among all questions asked.

Moreover, given the design and related data entry process (i.e., sequential data entry for a combination of multiple answer options and noting down and correcting the free text), distinguishing between answers, answer combinations, and corrections might not always be straightforward. Nonetheless, we suggest that these types of questions get additional attention during training sessions, with more time being allocated to explaining and practising asking these questions, with additional guidance and supervision provided during the fieldwork. One could also consider limiting even further these types of questions in household surveys.

We also developed and tested a new classification of answer correction types. We found this classification useful and suggest it for future studies. For example, we found that almost 90% of all corrections were simple mistake corrections (which is less worrisome) or impulsive and repetitive answers (most likely due to non-responsive screen); hence, they should not be considered problematic. This suggested that the reasons for the remaining multiple zigzag corrections (around 10%) lie elsewhere. We speculate that the remaining corrections were made following additional clarifications or confirmations of previous or later answers (Table 1). However, to verify our assumptions and identify other factors that affect correction probability and frequency (e.g., the exact wording or any other contextual factors) and even further unpack reasons behind answer corrections, field observations and qualitative interviews with both respondents and interviewers will be necessary. This once again underlines the importance of adequate timing dedicated to the data collection training, extensive field questionnaire testing, and effective supervision and guidance.

We believe that our proposed classification of answer correction types accounts for several dimension of the answer correction process. However, we would like to invite other researchers to evaluate our classification, improve and optimise it further, and test its usefulness and applicability in other types of surveys and research settings. Using our survey data, we identified only five answer correction types (including no corrections), lacking missing or incomplete answers after correction. This is primarily due to a lack of ‘missing’ (or empty in content) timestamped entries in our paradata dataset (which by default is not possible) and our decision not to treat ‘Do not know’ answers as ‘missing’ entries. However, we also much acknowledge effective training and comprehensive training manuals, diligent work of our data collectors, and their dedication to prompt interviewees and complete all relevant fields, which we believe aspired minimisation of any missing data. About a third of all questions in our questionnaire had inbuilt error notification, prompts, and warnings. Our results suggest that such notifications are effective since the proportion of corrected questions was 3.3% higher for questions with notifications compared to those without them, and having notifications was significantly associated with the probability of making corrections but negatively associated with the number of such corrections. Additionally, Survey Solutions application had a built-in colour coding indicating survey section completion (red for incomplete and unanswered questions and green for complete), which prompted data collectors to answer all questions. For example, during some training sessions at several data collection sites, interviewers were insisting on learning how to achieve completeness ‘having all sections colour-coded as green’ in all survey sections and were ‘somewhat unhappy’ to finish the exercise with one or more sections remaining incomplete (or red).

### Strength and limitations

Given our focus on corrections during interviewing, in our analysis, we excluded implausible and impractical values (over 180 min for section completion duration) but allowed for multiple corrections during the module completion and switching between questions and questionnaire sections. We assumed that such restrictions could provide meaningful and practical insights into face-to-face data collection process, even if it would exclude and not account for long breaks in the interviews (stopped and resumed several days/weeks/months later) or other errors in timestamps (e.g., resulting from a change of a tablet’s calendar set up from local form to the Gregorian calendar during data collection). However, we recognise that this decision could be considered as one of the limitations of the study, as it potentially did not capture corrections based on office data quality and error checks (following which questionnaires were returned and/or reassigned back to the interviewer for correction in-field). Moreover, in our analyses, we specifically focused only on a subset of paradata that related to answers and corrections. We did not utilise the data with timestamped events that related to process-related activities (e.g., interviewer or supervisor comments; enabling and disabling questions; declaring answers as valid or invalid based on the passing or failing of programmed validation rules; switching between the questionnaire’s translations; recalculating system variable values based on manual correction), which could be considered another limitation of the study. As paradata were not readily available for export at the beginning of our data collection (due to software limitations), we did not evaluate individual and team productivity (e.g., average hours per contact attempt, contact attempts without success, number of interviews per workday), or estimate the response likelihood and perform measurement error evaluation [6]. However, we relied on experience from our local data collection teams and invested additional time into training and field testing.

### Research gaps for improving measurements of MNCH indicators in household surveys

Given a lack of other studies that have used paradata in MNCH field, we cannot compare our findings directly to other studies, and we would like to stimulate the wider use of survey paradata to advance survey design and implementation for collecting information on pregnancies and births and for other purposes.

Paradata provide a wealth of information and could augment surveys, particularly overseeing the data collection process. Not surprisingly, it has already found use in other health and medical areas. For example, similar to our study, paradata were previously used in telemedicine research to estimate time spent to complete a questionnaire [20] and to examine completion and impact of push notifications on data completion in behaviour risk assessment [21]. Other applications in health include examining the role of paradata in non-response adjustment process [22], underreporting errors and finding suggestions for methodological improvement for future surveys [23] and examining response time at the level of individual questions [24]. Other studies examined practical use of paradata, for example, as an interactive web-based data visualisation tool, providing survey staff with the information to monitor data collection daily [25]. Using paradata (along with metadata and embedded data) can also improve response rates, identify bias, and give a possible explanation for apparent outlier responses, providing an efficient method of conducting web-based Delphi surveys [26]. Overall, using paradata in health research suggests that paradata could be valuable in quantifying recruitment efforts and aid the development and evaluation of new recruitment strategies [27].

Future analyses could investigate the relationship between correction rates and correction type and being supervised by a supervisor or other colleagues, which potentially could prompt additional corrections under peer pressure. Other potential uses of paradata in MNCH research could include effort indicators, tracking individual and team productivity, estimating contact attempts without success, and response propensity. Outcome indicators and case status indicators can also include non-interviews by type and refusal patterns by respondent characteristics. Paradata in MNCH research can also be used to generate a dashboard/monitoring system or a validation system for collected data against external sources of information, hence, automatically flagging incorrect entries in the interviews.

## Conclusion

Accurate estimation of coverage indicators from household surveys is vital but contingent on data quality; hence, a better understanding of how to improve the questionnaire design and survey implementation is crucial. Paradata have the potential to enhance survey design and implementation for collecting information on pregnancies and births, leading to improved metrics of measurement in maternal and newborn health research. They can help to identify questions and sections with multiple corrections and shed light on typically hidden challenges in the survey’s content, process, and administration. Overall, our experience suggests that given the size of paradata and their complex structure, analysis is not always straightforward, and consideration should be given to the additional data management and programming skills required. Nonetheless, paradata provide a wealth of data, can improve the process of data collection using live survey monitoring, and can add value in improving survey data quality as well as efficiency.

## Supplementary Information

**Additional file 1.** Detailed overview of questions in Section 2 in FPH and FBH+.

**Additional file 2.** Example of survey paradata structure.

**Additional file 3.** STROBE guidelines checklist.

**Additional file 4.** Types of questions in Section 2 in FPH and FBH+.

**Additional file 5.** Detailed overview of questions correction by question type, content and structure and reproductive module.

**Additional file 6.** Correction patterns, 23 groups by the number of corrections per question.

**Additional file 7.** Ethical approval of local Institutional Review Boards.

## Data Availability

Data sharing and transfer agreements were jointly developed and signed by all collaborating partners. The datasets generated during the current study are deposited online at 10.17037/DATA.00001556 with data access subject to approval by collaborating parties.

## Abbreviations

CAPI: Computer-assisted personal interviewing
CAWI: Computer-assisted web interviewing
DHS: Demographic and Health Surveys
EN-INDEPTH: Every Newborn International Network for the Demographic Evaluation of Populations and Their Health
FBH+: Full birth history (+ denotes additional questions on pregnancy losses)
FPH: Full pregnancy history
HDSS: Health and Demographic Surveillance System
LMIC: Low- and middle-income countries
MICS: Multiple Indicator Cluster Survey
MNCH: Maternal, newborn, and child health
TOP: Termination of pregnancy
