# Supplementary material for: Paradata analyses to inform population-based survey capture of pregnancy outcomes: EN-INDEPTH study
Source: Popul Health Metr. 2021 Feb 8;19(Suppl 1):10. doi: 10.1186/s12963-020-00241-0 (PMC7869213; doi:10.1186/s12963-020-00241-0)
Supplement: Supplementary file 1 — Additional file 1. Detailed overview of questions in Section 2 in FPH and FBH+. [file 12963_2020_241_MOESM1_ESM.docx]

**Additional file 1:** **Detailed overview of questions in Section 2 in FPH and FBH+**

| **FPH module** | | | | |
| --- | --- | --- | --- | --- |
| **Section** | **Question** | **Question type** | **Question nature1** | **Question structure** |
| Section 2.1 Pregnancy History | P.200A Are you currently pregnant? | Categorical: Single-select | regular | no notification |
| Section 2.1 Pregnancy History | P.200AA Is this your first pregnancy? | Categorical: Single-select | regular | no notification |
| Section 2.1 Pregnancy History | P.200AAA Have you ever been pregnant? | Categorical: Single-select | regular | no notification |
| Section 2.1 Pregnancy History | P.201 First I would like to ask about all the live births you have had during your life. Have you ever given birth? | Categorical: Single-select | regular | no notification |
| Section 2.1 Pregnancy History | P.202 Do you have any sons or daughters to whom you have given birth who are now living with you? | Categorical: Single-select | regular | no notification |
| Section 2.1 Pregnancy History | P.203 How many sons live with you? | Numerical computational | regular | notification |
| Section 2.1 Pregnancy History | P.203 And how many daughters live with you? | Numerical computational | regular | notification |
| Section 2.1 Pregnancy History | P.204 Do you have any sons or daughters to whom you have given birth who are alive but do not live with you? | Categorical: Single-select | regular | no notification |
| Section 2.1 Pregnancy History | P.205 How many sons are alive but do not live with you? | Numerical computational | regular | notification |
| Section 2.1 Pregnancy History | P.205 And how many daughters are alive but do not live with you? | Numerical computational | regular | notification |
| Section 2.1 Pregnancy History | P.206 Have you ever given birth to a boy or girl who was born alive but later died?<br><br> | Categorical: Single-select | death | no notification |
| Section 2.1 Pregnancy History | P.207 How many boys have died? | Numerical computational | death | notification |
| Section 2.1 Pregnancy History | P.207 And how many girls have died? | Numerical computational | death | notification |
| Section 2.1 Pregnancy History | P.207AA Women sometimes have pregnancies that do not result in a live born child. That is, a pregnancy can end in a miscarriage, abortion or the child can be born dead. Have you ever had a pregnancy that did not end in a live birth? | Categorical: Single-select | death | notification |
| Section 2.1 Pregnancy History | P.207BB How many pregnancies have you had that did not end in a live birth? | Numerical computational | death | notification |
| Section 2.1 Pregnancy History | Please sum answer to questions and enter total (sons/daughters living, not living, died) | Numerical computational | regular | no notification |
| Section 2.1 Pregnancy History | P.209 Just to make sure that I have this right: Excluding your current pregnancy you have had in TOTAL %p208total% pregnancies during your life. Is that correct? | Categorical: Single-select | regular | no notification |
| Section 2.1 Pregnancy History | P.209B Just to make sure that I have this right: You have had in TOTAL %p208total% pregnancies during your life. Is that correct? | Categorical: Single-select | regular | no notification |
| Section 2.2 Reproduction Roster | P.211 Now I would like to record the names of all your pregnancies, whether born alive, born dead, or lost before full term, starting with the first one you had | System listing - text | regular | no notification |
| Section 2.2 Reproduction Roster | P.212A Was this %rostertitle% pregnancy a twin? | Categorical: Single-select | regular | no notification |
| Section 2.2 Reproduction Roster | P.212B Was the %rostertitle% born alive, born dead, or lost before birth? | Categorical: Single-select | death | no notification |
| Section 2.2 Reproduction Roster | P.212C Did that %rostertitle% cry, move, or breathe when it was born? | Categorical: Single-select | regular | no notification |
| Section 2.2 Reproduction Roster | P.213 Is <font color="blue">%rostertitle%</font> a boy or a girl? | Categorical: Single-select | regular | no notification |
| Section 2.2 Reproduction Roster | P.215d Day when born | Numerical computational | regular | notification |
| Section 2.2 Reproduction Roster | P.215m Month when born | Categorical: Single-select | regular | no notification |
| Section 2.2 Reproduction Roster | P.215y Year when was born | Numerical computational | regular | notification |
| Section 2.2 Reproduction Roster | P.215a How many months did your pregnancy with %rostertitle% last? | Numerical computational | regular | notification |
| Section 2.2 Reproduction Roster | P.216 Is <font color="blue">%rostertitle%</font> still alive? | Categorical: Single-select | regular | no notification |
| Section 2.2 Reproduction Roster | P.217 How old was <font color="blue">%rostertitle%</font> at <font color="blue">%rostertitle%</font> 's last birthday? | Numerical computational | regular | notification |
| Section 2.2 Reproduction Roster | P.220 How old was <font color="blue">%rostertitle%</font> when (he/she) died? | Categorical: Single-select | death | no notification |
| Section 2.2 Reproduction Roster | P.220d DAYS | Numerical computational | death | notification |
| Section 2.2 Reproduction Roster | P.220m MONTHS | Numerical computational | death | notification |
| Section 2.2 Reproduction Roster | P.220y YEARS | Numerical computational | death | notification |
| Section 2.2 Reproduction Roster | P.220AA_d DAY | Numerical computational | death | notification |
| Section 2.2 Reproduction Roster | P.220AA_m MONTH | Categorical: Single-select | death | no notification |
| Section 2.2 Reproduction Roster | P.220AA_y YEAR | Numerical computational | death | notification |
| Section 2.2 Reproduction Roster | P.220AB_d DAY | Numerical computational | death | notification |
| Section 2.2 Reproduction Roster | P.220AB_m MONTH | Categorical: Single-select | death | no notification |
| Section 2.2 Reproduction Roster | P.220AB_y YEAR | Numerical computational | death | notification |
| Section 2.2 Reproduction Roster | P.220AC How many months did this pregnancy last? | Numerical computational | death | notification |
| Section 2.2 Reproduction Roster | P.220AC_2 Did you want to get pregnant at that time? | Categorical: Single-select | regular | no notification |
| Section 2.2 Reproduction Roster | P.220AD Did you or someone else do something to end this pregnancy? | Categorical: Single-select | TOP | no notification |
| Section 2.2 Reproduction Roster | P.220AD_A Was it a boy or a girl? | Categorical: Single-select | regular | no notification |
| Section 2.2 Reproduction Roster | P.221 Were there any other pregnancies between the previous pregnancy and <font color="blue">%rostertitle%</font>? | Categorical: Single-select | regular | no notification |
| Section 2.2 Reproduction Roster | P.222 Have you had any pregnancies since <font color="blue">%rostertitle%</font>? | Categorical: Single-select | regular | no notification |
| Section 2.2 Reproduction Roster | P.223 Is the above data correct? | Categorical: Single-select | regular | no notification |
| Section 2.2 Reproduction Roster | P.223d We would like to get more information on the circumstances around pregnancy losses and the deaths of young babies to help understand the impact of these deaths and how to prevent them. We may like to come back and talk with you about your loss. Is this okay? | Categorical: Single-select | death | no notification |
| Section 2.3 Reproduction | P.227 How many months pregnant are you? MONTHS | Numerical computational | regular | notification |
| Section 2.3 Reproduction | P.228 When you got pregnant, did you want to get pregnant at that time? | Categorical: Single-select | regular | no notification |
| Section 2.3 Reproduction | P.229a Did you want to have a baby later on or did you not want any more children? | Categorical: Single-select | TOP | no notification |
| Section 2.3 Reproduction | P.229b Did you want to have a baby later on or did you not want any children? | Categorical: Single-select | TOP | no notification |
| Section 2.3 Reproduction | P.229_0 Have you ever had a period that was more than one week late? | Categorical: Single-select | TOP | no notification |
| Section 2.3 Reproduction | P.229_0BD Have you ever heard of Menstrual Regulation? | Categorical: Single-select | TOP | no notification |
| Section 2.3 Reproduction | P.229_1 Did you do anything to resume your period? | Categorical: Single-select | TOP | no notification |
| Section 2.3 Reproduction | P.229_1BD Have you ever used Menstrual Regulation? | Categorical: Single-select | TOP | no notification |
| Section 2.3 Reproduction | P.229_2 What did you do to resume your period? | Categorical: Single-select | TOP | no notification |
| Section 2.3 Reproduction | P.229_2o Please specify | Short free text | TOP | no notification |
| Section 2.3 Reproduction | P.229_2BD In the last five years did you use Menstrual Regulation? | Categorical: Single-select | TOP | no notification |
| Section 2.3 Reproduction | P.229_3 Where did you go to get help to get your period back? | Categorical: Single-select | TOP | no notification |
| Section 2.3 Reproduction | Specify (non-governmental organisation) | Short free text | TOP | no notification |
| Section 2.3 Reproduction | P.229_3K Where did you go to get help to get your period back? | Categorical: Single-select | TOP | no notification |
| Section 2.3 Reproduction | Specify (non-governmental organisation) | Short free text | TOP | no notification |
| Section 2.3 Reproduction | P.229_3BD Where did you use Menstrual Regulation the last time? | Categorical: Single-select | TOP | no notification |
| Section 2.3 Reproduction | Specify (non-governmental organisation) | Short free text | TOP | no notification |
| Section 2.3 Reproduction | P.229AA It is not uncommon for a woman to get pregnant at a time when circumstance would make it difficult to have a child. Have you ever gotten pregnant at a time when it would have been difficult for you to have a child, or when you did not want to have one? | Categorical: Single-select | TOP | no notification |
| Section 2.3 Reproduction | P.229AB Did you or anyone else ever successfully do anything to end that pregnancy? | Categorical: Single-select | TOP | no notification |
| Section 2.3 Reproduction | P.229AC Did you have such a pregnancy in the last five years? | Categorical: Single-select | TOP | no notification |
| Section 2.3 Reproduction | P.229B IF SHE HAS HAD AN ABORTION IN THE LAST 5 YEARS: What were the reasons you decided to have this (last) abortion? | Categorical: Multi-select | TOP | no notification |
| Section 2.3 Reproduction | P.229BK IF SHE HAS HAD AN ABORTION IN THE LAST 5 YEARS: What were the reasons you decided to have this (last) abortion? | Categorical: Multi-select | TOP | no notification |
| Section 2.3 Reproduction | P.229Bo Please specify | Short free text | TOP | no notification |
| Section 2.3 Reproduction | P.229BB What was the MAIN reason you decided to have this (last) abortion? | Categorical: Single-select | TOP | no notification |
| Section 2.3 Reproduction | P.229BBo Please specify | Short free text | TOP | no notification |
| Section 2.3 Reproduction | P.229C What did you do to end this pregnancy? | Categorical: Single-select | TOP | no notification |
| Section 2.3 Reproduction | P.229Co Please specify | Short free text | TOP | no notification |
| Section 2.3 Reproduction | P.229Co Please specify | Categorical: Single-select | TOP | no notification |
| Section 2.3 Reproduction | P.229Do Please specify | Short free text | TOP | no notification |
| Section 2.3 Reproduction | P.229E Who did you see to get this done? | Categorical: Single-select | TOP | no notification |
| Section 2.3 Reproduction | P.229Eo Please specify | Short free text | TOP | no notification |
| Section 2.3 Reproduction | P.229F Where did you go to get this done? | Categorical: Single-select | TOP | no notification |
| Section 2.3 Reproduction | P.229Fo4 Please specify (primary healthcare centre) | Short free text | TOP | no notification |
| Section 2.3 Reproduction | P.229Fo7 Please specify (other health facility) | Short free text | TOP | no notification |
| Section 2.3 Reproduction | P.229Fo8 Please specify (non-governmental organisation) | Short free text | TOP | no notification |
| Section 2.3 Reproduction | P.229Fo9 Please specify (private hospital) | Short free text | TOP | no notification |
| Section 2.3 Reproduction | P.229Fo10 Please specify (private clinic) | Short free text | TOP | no notification |
| Section 2.3 Reproduction | P.229Fo11 Please specify (other private health institution) | Short free text | TOP | no notification |
| Section 2.3 Reproduction | P.229G How much did you pay for this abortion, including gifts or money given to the doctor (or person who performed this abortion)? | Categorical: Single-select | TOP | no notification |
| Section 2.3 Reproduction | P.229Gam Please specify the amount | Numerical computational | TOP | no notification |
| Section 2.3 Reproduction | P.239d Is the date available? | Categorical: Single-select | regular | no notification |
| Section 2.3 Reproduction | P.239 When did your last menstrual period start? | Date | regular | no notification |
| Section 2.3 Reproduction | P.239 If date was not given wasn't clear, please specify | Categorical: Single-select | regular | no notification |
| Section 2.3 Reproduction | P.239a_d Days ago | Numerical computational | regular | notification |
| Section 2.3 Reproduction | P.239a_w Weeks ago | Numerical computational | regular | notification |
| Section 2.3 Reproduction | P.239a_w Months ago | Numerical computational | regular | notification |
| Section 2.3 Reproduction | P.239a_y Years ago | Numerical computational | regular | notification |
| Section 2.3 Reproduction | P.240 From one menstrual period to the next, are there certain days when a woman is more likely to become pregnant? | Categorical: Single-select | regular | no notification |
| Section 2.3 Reproduction | P.241 Is this time just before her period begins, during her period, right after her period has ended, or halfway between two periods? | Categorical: Single-select | regular | no notification |
| Section 2.3 Reproduction | P.241 Please SPECIFY | Short free text | regular | no notification |
| Section 2.3 Reproduction | P.242 After the birth of a child, can a woman become pregnant before her menstrual period has returned? | Categorical: Single-select | regular | no notification |

| **FBH module** | | | | |
| --- | --- | --- | --- | --- |
| **Section** | Question | **Question type** | **Question nature1** | **Question structure** |
| Section 2.1 Birth History | Q.201 Now I would like to ask about all the births you have had during your life. Have you ever given birth? | Categorical: Single-select | regular | no notification |
| Section 2.1 Birth History | Q.202 Do you have any sons or daughters to whom you have given birth who are now living with you? | Categorical: Single-select | regular | no notification |
| Section 2.1 Birth History | Q.203 How many sons live with you? | Numerical computational | regular | notification |
| Section 2.1 Birth History | Q.203 And how many daughters live with you? | Numerical computational | regular | notification |
| Section 2.1 Birth History | Q.204 Do you have any sons or daughters to whom you have given birth who are alive but do not live with you? | Categorical: Single-select | regular | no notification |
| Section 2.1 Birth History | Q.205 How many sons are alive but do not live with you? | Numerical computational | regular | notification |
| Section 2.1 Birth History | Q.205 And how many daughters are alive but do not live with you? | Numerical computational | regular | notification |
| Section 2.1 Birth History | Q.206 Have you ever given birth to a boy or girl who was born alive but later died? | Categorical: Single-select | death | no notification |
| Section 2.1 Birth History | Q.207 How many boys have died? | Numerical computational | death | notification |
| Section 2.1 Birth History | Q.207 And how many girls have died? | Numerical computational | death | notification |
| Section 2.1 Birth History | Q.209 Please sum answer to questions and enter total (sons/daughters living, not living, died) | Numerical computational | regular | no notification |
| Section 2.1 Birth History | Q.209a Just to make sure that I have this right: you have had in TOTAL %q209% births during your life. Is that correct? | Categorical: Single-select | regular | no notification |
| Section 2.2 Reproduction roster | Q.211 Now I would like to record the names of all your births, whether still alive or not, starting with the first one you had. What name was given to your (first/next) baby? | System listing - text | regular | no notification |
| Section 2.2 Reproduction roster | Q.213 Is %rostertitle% a boy or a girl? | Categorical: Single-select | regular | no notification |
| Section 2.2 Reproduction roster | Q.214 Was %rostertitle% a twin? | Categorical: Single-select | regular | no notification |
| Section 2.2 Reproduction roster | Q.215d Day when born | Numerical computational | regular | notification |
| Section 2.2 Reproduction roster | Q.215m Month when born | Categorical: Single-select | regular | no notification |
| Section 2.2 Reproduction roster | Q.215y Year when born | Numerical computational | regular | notification |
| Section 2.2 Reproduction roster | Q.215a How many months did your pregnancy with %rostertitle% last? | Numerical computational | regular | notification |
| Section 2.2 Reproduction roster | Q.216 Is <font color="blue">%rostertitle%</font> still alive? | Categorical: Single-select | regular | no notification |
| Section 2.2 Reproduction roster | Q.217 How old was <font color="blue">%rostertitle%</font> at <font color="blue">%rostertitle%</font>'s last birthday? | Numerical computational | regular | notification |
| Section 2.2 Reproduction roster | Q.218 Is <font color="blue">%rostertitle%</font> living with you? | Categorical: Single-select | regular | no notification |
| Section 2.2 Reproduction roster | Q.220 How old was %rostertitle% when (he/she) died? | Categorical: Single-select | death | no notification |
| Section 2.2 Reproduction roster | Q.220d DAYS | Numerical computational | death | notification |
| Section 2.2 Reproduction roster | Q.220m MONTHS | Numerical computational | death | notification |
| Section 2.2 Reproduction roster | Q.220y YEARS | Numerical computational | death | notification |
| Section 2.2 Reproduction roster | Q.221 Were there any other live births before <font color="blue">%rostertitle%</font>, including any children who died after birth? | Categorical: Single-select | regular | no notification |
| Section 2.2 Reproduction roster | Q.221 Were there any other live births between <font color="blue">%prev_name%</font> and <font color="blue">%rostertitle%</font>, including any children who died after birth? | Categorical: Single-select | regular | no notification |
| Section 2.2 Reproduction roster | Q.222 Have you had any live births since the birth of <font color="blue">%rostertitle%</font>? | Categorical: Single-select | regular | no notification |
| Section 2.2 Reproduction roster | Q.223 Is the above data correct? | Categorical: Single-select | regular | no notification |
| Section 2.3 Reproduction | Q.226 Are you pregnant now? | Categorical: Single-select | regular | no notification |
| Section 2.3 Reproduction | Q.227 How many months pregnant are you? | Numerical computational | regular | notification |
| Section 2.3 Reproduction | Q.228 When you got pregnant, did you want to get pregnant at that time? | Categorical: Single-select | regular | no notification |
| Section 2.3 Reproduction | Q.229a Did you want to have a baby later on or did you not want any more children? | Categorical: Single-select | regular | no notification |
| Section 2.3 Reproduction | Q.229b Did you want to have a baby later on or did you not want any children? | Categorical: Single-select | regular | no notification |
| Section 2.3 Reproduction | Q.230 Have you ever had a pregnancy that miscarried, was aborted, or ended in a stillbirth? | Categorical: Single-select | death | no notification |
| Section 2.3 Reproduction | Q.231 When did such last pregnancy end? MONTH | Categorical: Single-select | death | no notification |
| Section 2.3 Reproduction | Q.231 When did such last pregnancy end? YEAR | Numerical computational | death | no notification |
| Section 2.3 Reproduction | Q.234 How many MONTHs pregnant were you when that pregnancy ended? | Numerical computational | death | notification |
| Section 2.3 Reproduction | Q.235 Since January 2012, have you had any other pregnancies that did not result in a live birth? | Categorical: Single-select | death | no notification |
| Section 2.3 Reproduction | Q.233_2m When did such pregnancy end? MONTH | Categorical: Single-select | death | no notification |
| Section 2.3 Reproduction | Q.233_2y When did such pregnancy end? YEAR | Numerical computational | death | notification |
| Section 2.3 Reproduction | Q.234_2 How many MONTHs pregnant were you when that pregnancy ended? | Numerical computational | death | no notification |
| Section 2.3 Reproduction | Q.235_2 Since January 2012, have you had any other pregnancies that did not result in a live birth? | Categorical: Single-select | death | no notification |
| Section 2.3 Reproduction | Q.233_3m When did such pregnancy end? MONTH | Categorical: Single-select | death | no notification |
| Section 2.3 Reproduction | Q.233_3y When did such pregnancy end? YEAR | Numerical computational | death | notification |
| Section 2.3 Reproduction | Q.234_3 How many MONTHs pregnant were you when that pregnancy ended? | Numerical computational | death | notification |
| Section 2.3 Reproduction | Q.235_3 Since January 2012, have you had any other pregnancies that did not result in a live birth? | Categorical: Single-select | death | no notification |
| Section 2.3 Reproduction | Q.233_4m When did such pregnancy end? MONTH | Categorical: Single-select | death | no notification |
| Section 2.3 Reproduction | Q.233_4y When did such pregnancy end? YEAR | Numerical computational | death | notification |
| Section 2.3 Reproduction | Q.234_4 How many MONTHs pregnant were you when that pregnancy ended? | Numerical computational | death | notification |
| Section 2.3 Reproduction | Q.235_4 Since January 2012, have you had any other pregnancies that did not result in a live birth? | Categorical: Single-select | death | no notification |
| Section 2.3 Reproduction | Q.237 Did you have any miscarriages, abortions or stillbirths that ended before 2012? | Categorical: Single-select | death | notification |
| Section 2.3 Reproduction | Q.238a When did the last such pregnancy that terminated before 2012 end? Please record the MONTH | Categorical: Single-select | death | no notification |
| Section 2.3 Reproduction | Q.238b Please record the YEAR | Numerical computational | death | notification |
| Section 2.3 Reproduction | Q.239d Is the date available? | Categorical: Single-select | regular | no notification |
| Section 2.3 Reproduction | Q.239st When did your last menstrual period start? | Date | regular | no notification |
| Section 2.3 Reproduction | Q.239a If date was not given or wasn't clear, please specify | Categorical: Single-select | regular | no notification |
| Section 2.3 Reproduction | Q.239a_d Days ago | Numerical computational | regular | notification |
| Section 2.3 Reproduction | Q.239a_w Weeks ago | Numerical computational | regular | notification |
| Section 2.3 Reproduction | Q.239a_m Months ago | Numerical computational | regular | notification |
| Section 2.3 Reproduction | Q.239a_y Years ago | Numerical computational | regular | notification |
| Section 2.3 Reproduction | Q.240 From one menstrual period to the next, are there certain days when a woman is more likely to become pregnant? | Categorical: Single-select | regular | no notification |
| Section 2.3 Reproduction | Q.241 Is this time just before her period begins, during her period, right after her period has ended, or halfway between two periods? | Categorical: Single-select | regular | no notification |
| Section 2.3 Reproduction | Q.241o If other, specify | Short free text | regular | no notification |
| Section 2.3 Reproduction | Q.242 After the birth of a child, can a woman become pregnant before her menstrual period has returned? | Categorical: Single-select | regular | no notification |

Notes: FPH - full pregnancy history module. FBH+ - full birth history module with additional questions on pregnancy losses.
