# Supplementary material for: Paradata analyses to inform population-based survey capture of pregnancy outcomes: EN-INDEPTH study
Source: Popul Health Metr. 2021 Feb 8;19(Suppl 1):10. doi: 10.1186/s12963-020-00241-0 (PMC7869213; doi:10.1186/s12963-020-00241-0)
Supplement: Supplementary file 2 — Additional file 2. Example of survey paradata structure. [file 12963_2020_241_MOESM2_ESM.docx]

**Additional file 2: Example of survey paradata structure**

| Interview_id | Order | Action | Responsible | Role | Parameters | Timestamp | Entry | Version | Code |
| --- | --- | --- | --- | --- | --- | --- | --- | --- | --- |
| 00004b398ca5495a993448236e4974ac | 49 | AnswerSet | IntN | Interviewer | p207aa | 18/05/2018 06:08 | 2 | 14.8 | Int42 |
| 00004b398ca5495a993448236e4974ac | 52 | AnswerSet | IntN | Interviewer | p208total | 18/05/2018 06:09 | 8 | 14.8 | Int42 |
| 00004b398ca5495a993448236e4974ac | 64 | AnswerSet | IntN | Interviewer | p215d | 18/05/2018 06:10 | 17 | 14.8 | Int42 |
| 00004b398ca5495a993448236e4974ac | 136 | QuestionDisabled | IntN | Interviewer | p217 | 18/05/2018 06:10 |  | 14.8 | Int42 |

Notes: Headings in the exported paradata file: Interiew_id – identifier of the interview affected by the evening, order – numerical sequential ID of the event, action – events recorded in the paradata, responsible – a person responsible for the event, role – the role of the person mentioned in the responsible column, parameters – question variables, timestamp – data and time when the event occurred combined in a single timestamp, entry – value entered, version – version of the questionnaire used, code – code of the responsible person. More detailed overview of possible actions reflected in the paradata can be found at: https://support.mysurvey.solutions/headquarters/export/paradata_file_format
