# Supplementary material for: Paradata analyses to inform population-based survey capture of pregnancy outcomes: EN-INDEPTH study
Source: Popul Health Metr. 2021 Feb 8;19(Suppl 1):10. doi: 10.1186/s12963-020-00241-0 (PMC7869213; doi:10.1186/s12963-020-00241-0)
Supplement: Supplementary file 4 — Additional file 4. Types of questions in Section 2 in FPH and FBH+. [file 12963_2020_241_MOESM4_ESM.docx]

**Additional file 4: Types of questions in Section 2 in FPH and FBH+**

|  | N unique questions | | N timestamped entries | | | | | |
| --- | --- | --- | --- | --- | --- | --- | --- | --- |
|  | FPH, n | FBH+, n | FPH, n | % | FBH+, n | % | Overall | % |
| **Total** | 98 | 66 | 1,891,728 | 100.0 | 1,735,643 | 100.0 | 3,627,371 | 100.0 |
| Section 2.1 History | 18 | 12 | 379,905 | 20.1 | 290,050 | 16.7 | 669,955 | 18.5 |
| Section 2.2 Roster | 29 | 18 | 1,240,476 | 65.6 | 1,160,200 | 66.8 | 2,400,676 | 66.2 |
| Section 2.3 Reproduction | 51 | 36 | 271,347 | 14.3 | 285,393 | 16.4 | 556,740 | 15.3 |
| **Question type** |  |  |  |  |  |  |  |  |
| Single-select | 52 | 35 | 1,274,905 | 67.4 | 1,141,269 | 65.8 | 2,416,174 | 66.6 |
| Multi-select | 2 | 0 | 375 | 0.0 | 0 | 0.0 | 375 | 0.0 |
| Numerical computational | 26 | 28 | 593,720 | 31.4 | 572,004 | 33.0 | 1,165,724 | 32.1 |
| Date-related | 1 | 1 | 22,282 | 1.2 | 22,321 | 1.3 | 44,603 | 1.2 |
| Free-text | 17 | 2 | 446 | 0.0 | 49 | 0.0 | 495 | 0.0 |
| **Question nature** |  |  |  |  |  |  |  |  |
| Regular | 41 | 39 | 1,555,229 | 82.2 | 1,609,107 | 92.7 | 3,164,336 | 87.2 |
| Death-related | 18 | 27 | 247,228 | 13.1 | 126,536 | 7.3 | 373,764 | 10.3 |
| TOP-related | 39 | 0 | 89,271 | 4.7 | 0 | 0.0 | 89,271 | 2.5 |
| **Question structure** |  |  |  |  |  |  |  |  |
| With notification | 25 | 26 | 593,294 | 31.4 | 533,761 | 30.8 | 1,127,055 | 31.1 |
| Without notification | 73 | 40 | 1,298,434 | 68.6 | 1,201,882 | 69.2 | 2,500,316 | 68.9 |

Notes: Totals for percentages might not always add up to 100% due to rounding and formatting. FPH - full pregnancy history module. FBH+ - full birth history module with additional questions on pregnancy losses.
