# Supplementary material for: Paradata analyses to inform population-based survey capture of pregnancy outcomes: EN-INDEPTH study
Source: Popul Health Metr. 2021 Feb 8;19(Suppl 1):10. doi: 10.1186/s12963-020-00241-0 (PMC7869213; doi:10.1186/s12963-020-00241-0)
Supplement: Supplementary file 5 — Additional file 5. Detailed overview of questions correction by question type, content and structure and reproductive module. [file 12963_2020_241_MOESM5_ESM.docx]

**Additional file 5: Detailed overview of questions correction by question type, content and structure and reproductive module**

|  | **Overall** | | | | | | | **FPH** | | | | | | | **FBH+** | | | | | | |
| --- | --- | --- | --- | --- | --- | --- | --- | --- | --- | --- | --- | --- | --- | --- | --- | --- | --- | --- | --- | --- | --- |
|  | **All questions** | | | | **Per corrected question** | | | **All questions** | | | | **Per corrected question** | | | **All questions** | | | | **Per corrected question** | | |
| **Question Type** | **N questions asked** | | **N questions with corrections** | | **Corrections** | | **Max corrections** | **N questions asked** | | **N questions with corrections** | | **Corrections** | | **Max corrections** | **N questions asked** | | **N questions with corrections** | | **Corrections** | | **Max corrections** |
|  | n | % | n | % | Mean (SD) | Median | N | n | % | n | % | Mean (SD) | Median | n | n | % | n | % | Mean (SD) | Median | n |
| **Type** |  |  |  |  |  |  |  |  |  |  |  |  |  |  |  |  |  |  |  |  |  |
| Single-select | 2,248,009 | 67.3 | 147,157 | 58.7 | 1.14(0.49) | 1 | 28 | 1,185,935 | 68.1 | 77,508 | 59.6 | 1.15(0.51) | 1 | 28 | 1,062,074 | 66.46 | 69,649 | 57.7 | 1.14(0.48) | 1 | 24 |
| Multi-select | 192 | 0.01 | 86 | 0.03 | 2.13 (2.33) | 1 | 19 | 192 | 0.01 | 86 | 0.07 | 2.13(2.33) | 1 | 19 | n/a | n/a | n/a | n/a | n/a | n/a | n/a |
| Numerical computational | 1,048,992 | 31.4 | 101,607 | 40.5 | 1.15(0.52) | 1 | 23 | 534,414 | 30.7 | 51,510 | 39.6 | 1.15(0.54) | 1 | 23 | 514,578 | 32.2 | 50,097 | 41.5 | 1.15(0.51) | 1 | 20 |
| Date-related | 42,582 | 1.3 | 1,691 | 0.7 | 1.19(0.64) | 1 | 8 | 21,262 | 1.2 | 841 | 0.6 | 1.21(0.70) | 1 | 8 | 21,320 | 1.33 | 850 | 0.7 | 1.(0.56) | 1 | 5 |
| Free-text | 414 | 0.01 | 67 | 0.03 | 1.21 (0.59) | 1 | 5 | 369 | 0.02 | 63 | 0.05 | 1.22(0.61) | 1 | 5 | 45 | 0 | 4 | 0 | 1(0) | 1 | 1 |
| **Nature** |  |  |  |  |  |  |  |  |  |  |  |  |  |  |  |  |  |  |  |  |  |
| Regular | 2,912,643 | 87.2 | 221,447 | 88.4 | 1.13(0.48) | 1 | 28 | 1,430,660 | 82.1 | 109,424 | 84.2 | 1.13(0.490 | 1 | 28 | 1,481,983 | 92.74 | 112,023 | 92.9 | 1.14(0.48) | 1 | 24 |
| Death-related | 346,067 | 10.4 | 22,628 | 9.03 | 1.22(0.68) | 1 | 23 | 230,033 | 13.2 | 14,051 | 10.8 | 1.22 (0.69) | 1 | 23 | 116,034 | 7.26 | 8,577 | 7.11 | 1.22(0.660 | 1 | 13 |
| TOP-related | 81,479 | 2.4 | 6,533 | 2.6 | 1.19(0.62) | 1 | 19 | 81,479 | 4.7 | 6,533 | 5.03 | 1.19(0.62) | 1 | 19 | n/a | n/a | n/a | n/a | n/a | n/a | n/a |
| **Structure** |  |  |  |  |  |  |  |  |  |  |  |  |  |  |  |  |  |  |  |  |  |
| Built-in error notification | 1,012,865 | 30.3 | 99,532 | 39.7 | 1.15(0.52) | 1 | 23 | 534,107 | 30.7 | 51,486 | 39.6 | 1.15(0.54) | 1 | 23 | 478,758 | 29.96 | 48,046 | 39.8 | 1.14(0.51) | 1 | 20 |
| No built-in error notification | 2,327,324 | 69.7 | 151,076 | 60.3 | 1.14(0.50) | 1 | 28 | 1,208,065 | 69.3 | 78,522 | 60.4 | 1.15(0.51) | 1 | 28 | 1,119,259 | 70.04 | 72,554 | 60.2 | 1.14(0.49) | 1 | 24 |
| **Overall** | 3,340,189 | 100 | 250,608 | 100 | 1.14 (0.51) | 1 | 28 | 1,742,172 | 52.2 | 130,008 | 7.5 | 0.15(0.52) | 1 | 28 | 1,598,017 | 47.8 | 120,600 | 7.5 | 1.14(0.49) | 1 | 24 |

Notes: FPH - full pregnancy history module. FBH+ - full birth history module with additional questions on pregnancy losses.
