# Supplementary material for: Paradata analyses to inform population-based survey capture of pregnancy outcomes: EN-INDEPTH study
Source: Popul Health Metr. 2021 Feb 8;19(Suppl 1):10. doi: 10.1186/s12963-020-00241-0 (PMC7869213; doi:10.1186/s12963-020-00241-0)
Supplement: Supplementary file 6 — Additional file 6. Correction patterns, 23 groups by the number of corrections per question. [file 12963_2020_241_MOESM6_ESM.docx]

**Additional file 6: Correction patterns, 23 groups by the number of corrections per question**

|  | Overall | | FPH | | FBH+ | |
| --- | --- | --- | --- | --- | --- | --- |
| N corrections | n | % | n | % | n | % |
| 1 | 222,922 | 89.0 | 115,365 | 88.7 | 107,557 | 89.2 |
| 2 | 22,064 | 8.8 | 11,590 | 8.9 | 10,474 | 8.7 |
| 3 | 3,976 | 1.6 | 2,132 | 1.6 | 1,844 | 1.5 |
| 4 | 967 | 0.4 | 526 | 0.4 | 441 | 0.4 |
| 5 | 363 | 0.1 | 197 | 0.2 | 166 | 0.1 |
| 6 | 130 | 0.1 | 89 | 0.1 | 41 | 0.0 |
| 7 | 69 | 0.0 | 45 | 0.0 | 24 | 0.0 |
| 8 | 37 | 0.0 | 22 | 0.0 | 15 | 0.0 |
| 9 | 25 | 0.0 | 17 | 0.0 | 8 | 0.0 |
| 10 | 13 | 0.0 | 5 | 0.0 | 8 | 0.0 |
| 11 | 11 | 0.0 | 5 | 0.0 | 6 | 0.0 |
| 12 | 9 | 0.0 | 4 | 0.0 | 5 | 0.0 |
| 13 | 6 | 0.0 | 1 | 0.0 | 5 | 0.0 |
| 14 | 3 | 0.0 | 3 | 0.0 | . | . |
| 16 | 2 | 0.0 | 1 | 0.0 | 1 | 0.0 |
| 17 | 2 | 0.0 | 1 | 0.0 | 1 | 0.0 |
| 18 | 1 | 0.0 | 1 | 0.0 | . | . |
| 19 | 3 | 0.0 | 2 | 0.0 | 1 | 0.0 |
| 20 | 1 | 0.0 | . | . | 1 | 0.0 |
| 22 | 1 | 0.0 | . | . | 1 | 0.0 |
| 23 | 1 | 0.0 | 1 | 0.0 | . | . |
| 24 | 1 | 0.0 | . | . | 1 | 0.0 |
| 28 | 1 | 0.0 | 1 | 0.0 | . | . |
| Total N groups | 23 | | 20 | | 19 | |
|  | n | % | n | % | n | % |
| N correction patterns | 250,608 | 100 | 130,008 | 51.9 | 120,600 | 48.1 |

Notes FPH - full pregnancy history module. FBH+ - full birth history module with additional questions on pregnancy losses.
